# The African cholera surveillance network (Africhol) consortium meeting, 10–11 June 2015, Lomé, Togo

**DOI:** 10.1186/s12919-016-0068-z

**Published:** 2017-01-31

**Authors:** Aline Munier, Berthe-Marie Njanpop-Lafourcade, Delphine Sauvageot, Raymond B. Mhlanga, Léonard Heyerdahl, Johara Nadri, Richard Wood, Issaka Ouedraogo, Alexandre Blake, Laurent Akilimali Mukelenge, Jean-Claude B. Anné, Abiba Banla Kere, Lucienne Dempouo, Sakoba Keita, José P. M. Langa, Issa Makumbi, Elibariki R. Mwakapeje, Ian J. Njeru, Olubunmi E. Ojo, Isaac Phiri, Lorenzo Pezzoli, Bradford D. Gessner, Martin Mengel

**Affiliations:** 1 Agence de Médecine Préventive, 21 bd Pasteur, 75015 Paris, France; 2 Agence de Médecine Préventive, Abidjan, Côte d’Ivoire; 3 Agence de Médecine Préventive, Ferney-Voltaire, France; 4 Agence de Médecine Préventive, Bobo-Dioulasso, Burkina Faso; 5 Institut National de Recherche Biomédicale, Ministère de la Santé Publique, Kinshasa, Democratic Republic of the Congo; 6 Institut Pasteur de Cote d’Ivoire, Centre National de Référence choléra et shigelloses, Abidjan, Côte d’Ivoire; 7 Institut National d’Hygiène, Lomé, Togo; 8 Ministère de la Santé, Yaoundé, Cameroon; 9 Ministère de la Santé, Conakry, Guinea; 10 National Institute of Health, Maputo, Mozambique; 11 Ministry of Health, National Disease Control, Kampala, Uganda; 12 Ministry of Health, Dar es Salaam, Tanzania; 13 Ministry of Health, Nairobi, Kenya; 14 Federal Ministry of Health, Abuja, Nigeria; 15 Ministry of Health and Child Welfare, Harare, Zimbabwe; 16 World Health Organization, Secretariat of the Global Task Force on Cholera Control (GTFCC), Geneva, Switzerland; 17 Current affiliation: Epicentre, Paris, France

**Keywords:** Cholera, Surveillance, Africa, Epidemiology, Outbreaks, Prevention, Control, Vaccination

## Abstract

The fifth annual meeting of the African cholera surveillance network (Africhol) took place on 10–11 June 2015 in Lomé, Togo. Together with international partners, representatives from the 11 member countries -Cameroon, Côte d’Ivoire, Democratic Republic of Congo, Guinea, Kenya, Mozambique, Nigeria, Tanzania, Togo, Uganda, Zimbabwe- and an invited country (Malawi) shared their experience. The meeting featured three sessions: i) cholera surveillance, prevention and control in participating countries, ii) cholera surveillance methodology, such as cholera mapping, cost-effectiveness studies and the issue of overlapping epidemics from different diseases, iii) cholera laboratory diagnostics tools and capacity building. The meeting has greatly benefitted from the input of technical expertise from participating institutions and the observations emerging from the meeting should enable national teams to make recommendations to their respective governments on the most appropriate and effective measures to be taken for the prevention and control of cholera.

Recommendations for future activities included collecting precise burden estimates in surveillance sites; modeling cholera burden for Africa; setting up cross-border collaborations; strengthening laboratory capacity for the confirmation of suspected cholera cases and for vaccine impact assessment in settings where oral cholera vaccine would be used; adapting cholera surveillance to concurrent issues (e.g., Ebola); and developing national cholera control plans including rationale vaccination strategies together with other preventive and control measures such as improvements in water, sanitation and hygiene (WASH).

**Electronic supplementary material:**

The online version of this article (doi:10.1186/s12919-016-0068-z) contains supplementary material, which is available to authorized users.

## The Africhol project

Cholera remains a substantial contributor to public health burden in many developing countries, particularly in sub-Sahara Africa. In 2014, 190,549 cases were reported to the World Health Organization (WHO), including 55% from Africa [1]. Of the 2,231 deaths reported worldwide, 1882 (84.4%) occurred in Africa. However, the true burden of cholera is poorly known and likely underestimated, because of limitations in current national surveillance systems including under-reporting, type of case-definitions used and lack of laboratory diagnostic capacities. An estimated 2.9 million cholera cases (1.3 m–4.0 m) and 95,000 cholera deaths (21,000-143,000) occur each year [2].

Targeting cholera prevention through increased access to water, sanitation and hygiene (WASH) and oral cholera vaccines (OCV) requires accurate epidemiological data at the local level. Further, such data help international organizations to prioritize where to support the places most in need.

The African Cholera Surveillance Network [3] was launched in 2009 as a consortium of organizations with expertise in cholera prevention and control and is managed and technically supported by the Agence de Médecine Préventive (AMP). It aims to generate data to enable national and international stakeholders determine the most appropriate interventions for prevention and control of cholera. Africhol supports countries by implementing a common protocol for data collection and analysis and building capacity for cholera diagnosis.

Study methods have been approved for each country by national ethical review boards or public-health authorities. The project has created a sustainable network for prospective cholera surveillance in high-incidence areas of 11 African countries: Cameroon, Côte d’Ivoire, Democratic Republic of the Congo (DRC), Guinea, Kenya, Mozambique, Nigeria, Tanzania, Togo, Uganda, and Zimbabwe. The National Institute for Communicable Diseases (NICD) in South Africa participates for laboratory quality control and genotyping. Africhol is part of the Global Task Force on Cholera Control (GTFCC) at WHO, which aims to support increased implementation of evidence-based strategies to control cholera [4].

The annual Africhol consortium meeting enables countries and partners to exchange on recent updates. The objectives of the fifth annual meeting, held in Lomé, Togo, in 10–11 June 2015, were to:

- Share the results of Africhol surveillance for 2011–2015;
- Identify trends in cholera epidemiology in Africa;
- Share experiences in the fight against cholera and promote dialogue and discussion;
- Strengthen country capacity including spatial data analysis and laboratory diagnostics.
- Apply results and experiences to cholera prevention interventions, including vaccine use.

Fifty-two participants attended, including 28 affiliated to Ministries of Health, 16 from international institutions and 8 from academic institutions (Additional file 1).

### Cholera surveillance, prevention and control in Africhol countries

According to the WHO standard case definition, a case of cholera should be suspected when:

- in an area where the disease is not known to be present, a patient aged 5 years or more develops severe dehydration or dies from acute watery diarrhea;
- in an area where there is a cholera epidemic, a patient aged 5 years or more develops acute watery diarrhea, with or without vomiting.

In each Africhol country, this definition was adapted and varied based on the project’s goals and the country preferences, including using a younger age threshold and less restrictive clinical criteria.

A case of cholera was considered confirmed when *Vibrio cholerae* O1 or O139 was isolated from a patient with diarrhea.

### Cholera epidemiology in Africhol countries 2011–2015

General patterns and local specificities of cholera epidemiology in Africhol countries emerged through the presentations and discussions, including:

- Age distribution: the majority of suspected cases were 15 to 59 years-old but a substantial proportion of suspected and confirmed cases were seen in children below age 5 years, especially in Goma, DRC. This highlights the lack of sensitivity of the WHO cholera case-definition used by most African countries that excludes persons under 5 years old. Zimbabwe, for example reacted after the 2008 cholera outbreak by changing the case definition to include cases 2 years and above.
- Symptomatology: over 80% of all cases had acute watery diarrhea, vomiting and severe dehydration. The distribution of clinical signs differed between countries. When applying the WHO case definition to the cases in our network, we found it to have high sensitivity but low specificity. For Africhol, we increased specificity by confirming all clinically suspected cases by laboratory culture whenever possible. Using Africhol data we are working to determine the specificity and sensitivity of various combinations of clinical signs showing that including cases age 2 years and above significantly increases sensitivity without decreasing specificity compared to the WHO case definition.
- Endemic vs. epidemic cholera: different epidemiological patterns for cholera disease were identified [5]. In certain countries such as the DRC, cases are reported throughout the year, especially in well delimited areas of hyper endemicity (i.e., “cholera hotspots”). Outbreaks may occur at irregular intervals in other countries, such as in Côte d’Ivoire, with an epidemic in 2014, or in Guinea where a very large outbreak occurred in 2012 with sporadic cases confirmed before and after this epidemic. In 2015, major epidemics occurred in East Africa associated with seasonal floods, in particular in Mozambique, Malawi, Zimbabwe, Kenya, and Tanzania.
- Identified groups at risk of cholera: according to the countries, geographic case clusters were observed, such as in specific neighborhoods of Lomé. Specific populations at risk also represented a large proportion of cholera cases identified:
  - Internally displaced persons (IDPs), refugees and slum dwellers: in Goma, DRC, higher cholera burden was seen during the military conflict, and successive waves of the epidemic followed population movements. After the dismantling of IDP camps in 2014, cases declined. In Cameroon in 2014, many cases concentrated on the border with Nigeria where conflicts were ongoing. In Kenya, large national outbreaks started in Nairobi slums. In Tanzania an outbreak occurred in Kigoma region in May 2015 in the camps for refugees and asylum seekers, claiming 4,487 cases and 31 deaths.
  - Fishing communities: for example, in Abidjan, Côte d’Ivoire, that may play a role in the start of epidemics and act as vectors in transmitting the disease to resident populations. In Uganda, outbreaks also occur regularly in fishing communities, such as in Namayingo district in 2014. Africhol has implemented anthropological studies to better understand specific population practices and acceptance of interventions, and to adapt prevention and education messages.
  - These high-risk groups usually shared the same characteristics regarding WASH, with a low proportion of households with latrine coverage and access to safe drinking water. Improved detection, diagnosis, and reporting, as already done in Africhol countries, will be critical to improve context-adapted WASH interventions [6].
- Cross-border transmission: cholera endemic areas or populations are often independent from national borders. Hence, cross-border transmission is a concern, because of population movements and characteristics of specific populations such as fishermen who frequently cross riverine, lacustrine, or marine borders for occupational reasons. Africhol has been instrumental in starting discussion between several of its member countries to set up cross-border surveillance. For example, Uganda with Kenya and DR Congo, or Guinea-Conakry and its neighbors (Sierra Leone) has occurred and, during an outbreak in Zimbabwe in 2015, discussions started with Mozambique and Malawi. Some of the cross-border affected countries have started to initiate a dialogue, either during regional meetings, specific cross-border meetings at national level or directly in the districts concerned, in particular discussing the possibility to exchange data from cholera line lists, and to implement common control measures during an outbreak. For countries developing or adapting their national plan for the control and prevention of cholera, cross-border collaboration was added as a specific component of the plan, highlighting the importance of this concern.

### Response to epidemics and experience with OCV

- From an international perspective
  The long-term solution for cholera control remains increasing access to safe drinking water, hygiene and improved sanitation infrastructures, provided the latter is adapted to the local context. However, employing OCV has proven effective in reactive campaign in Africa [7]. Further, OCV has recently been shown to have a protective efficacy for at least 5 years [8]. This opens the possibility for preventive use of OCV in locations that are historically at high risk of cholera, especially in sites with low access to care and where it is not feasible to improve health and sanitation conditions in the short term. OCV can serve as a short- and medium-term step in controlling cholera while longer term solutions are put in place or where such solutions are difficult to put in place, e.g., in migrant populations. In October 2009, the WHO Strategic Advisory Group of Experts on immunization (SAGE) recommended that OCV should be considered as a reactive strategy during outbreaks, in addition to the already recommended preventive use in endemic areas [9]. A new bi-valent killed whole cell vaccine was pre-qualified by WHO in 2011 and a stockpile was created in 2013, with an initial two million doses to be available mainly for epidemic response in low-income countries, which has since expanded thanks to funding from Gavi, the Vaccine Alliance. In this context, more governments might consider cholera vaccination where needed [10]. Countries wishing to use the vaccine can request doses through two different mechanisms: 1) for emergency situations (i.e., responding to cholera outbreaks or humanitarian crises), the vaccine stockpile is managed by the International Coordinating Group (ICG) and 2) for non-emergency situations (i.e., conducting preventive vaccination campaigns in cholera hotspots) OCV is managed by the OCV Working Group of the GTFCC.
- From a national perspective

In 2015, two countries had different experiences requesting OCV doses in response to a cholera epidemic:

- In Malawi, floods affected 15 districts in the country in January 2015, causing the displacement of 230,000 people living in temporary camps. In February, a cholera outbreak started in the community in the Nsanje district, one of the most affected by the floods; this outbreak accounted for 58 of 78 suspected cholera cases nationally during this period, and was associated with two deaths. To prevent the spread of cholera to the IDP camps and the surrounding community, in March 2015 a request was submitted to the ICG and approved. The campaign in Nsanje targeted 160,000 people living in the IPD camps and the surrounding community. The campaign’s strengths included: very high administrative coverage in the first round (98%); availability of resources (vaccines, transport and cold chain); and strong motivation of health personnel and community leaders. Challenges included difficulties in administering the second dose, with 70% coverage (bad taste and smell, movement of the initially vaccinated populations); increased workload for health workers; and misinformation (people presenting for the first injection at the second round).
- Mozambique also faced a large epidemic in January-April 2015, when heavy rainfall was associated with more than 7,000 suspected cases and 59 deaths in five provinces together, including 1453 cases and 14 deaths in Zambezia province alone. The country decided to undertake an OCV campaign, whose goal was to prevent epidemic spread to the town of Quelimane (an important trade route) and the IDP camps in Zambezia province, which had limited access to clean water and latrines. A request for 181,496 OCV doses was submitted to the ICG in April 2015. The ICG declined the request arguing that a reactive campaign would have had a limited impact because the cholera epidemic was already decreasing in all affected areas including in the districts targeted for vaccination. This experience shows how important it is to continuously collect and use surveillance data and to have a clear strategy in place for OCV use, ideally incorporated into a national cholera control plan, and to anticipate administrative and logistical constraints, as well as the role of the national regulatory agency, to plan timely vaccination strategies.

## Cholera surveillance methodology

### Overlapping epidemics from different diseases: Ebola and cholera

Ebola has affected cholera-prone countries, including Guinea which is part of the Africhol consortium. Following the challenges for the health system, e.g., the loss of staff to disease and death, the threat of overlapping simultaneous epidemics is imminent. Therefore we have adapted cholera surveillance as follows:

- Extension of Africhol enhanced surveillance to coastal areas at high risk for the onset of cholera epidemics (which are also sites affected by Ebola in 2014–2015).
- Digital reporting in one pilot prefecture for instant data accessibility from prefecture to central level.
- No stool samples collected or tested to avoid risk of contamination by Ebola virus, as instructed by the Ministry of Health. This lack of case confirmation could have led to false cholera notifications due to diarrhea from other etiologies. To prevent this, we used an adapted and simplified case-report form mainly focusing on signs more specific to Ebola to distinguish cholera from Ebola. These signs included the presence of unexplained bleeding, unconsciousness, fever, and contact with an Ebola case.

Issues around both diseases can be dealt with through an integrated approach:

- Given the difficulties in monitoring and confirming both diseases and their overlapping case-definitions, a different case definition for cholera could be considered that might, for example, include the absence of fever as a way of distinguishing it from Ebola.
- The Ebola epidemic may have resulted in increased hygiene education campaigns and training of community members to detect signs of Ebola (and potentially cholera), which in turn may have made communities more resilient.
- Both diseases need strengthened surveillance including improved laboratory capacity in optimal safety conditions. This includes developing enhanced rapid Ebola tests; possibly performing cholera rapid diagnostic tests (RDTs) on stools that have been rendered safe through chlorination, radiation, or other measures; and considering the duration of Ebola virus shedding in stool.
- The use of OCV could be considered preemptively in populations at high risk of cholera and Ebola [11], potentially together with an Ebola vaccine that may be available in the future.

### Cholera risk mapping in Africa

Work on the dynamics of cholera in Africa by Johns Hopkins Bloomberg School of Public Health (Lessler, Azman et al.) used a large variety of official and unofficial reporting sources to detect regions with strong regular transmission compared to irregular transmission and to evaluate areas or hotspots where health interventions would have the most impact. In DRC, for example, cholera circulates endemically at a moderate incidence level but with very high regional differences, with 9% of the population living in areas where the incidence is higher than 1/1000.

For further analyses, it will be important to include detailed information on fatality ratios, as many countries that do not systematically report deaths may have the highest case fatality ratios.

### Cost-effectiveness of immunization strategies

In a context of limited resources, the use of cost-effectiveness models is of particular interest in helping decision-makers choose optimal intervention strategies. The cost-effectiveness of various OCV immunization strategies was presented by the representative of Fred Hutchinson Cancer Research Center, Seattle, USA. Using the examples of Goma (DRC), Bangladesh and Haiti, it showed that targeting different specific population groups (in terms of age, incidence of the disease) will affect the impact and, therefore, be more or less cost effective. In addition, the costs and benefits of interventions may differ depending on the implementing entity (government, private sector, communities). More complex models may take into account herd immunity and compare different strategies (e.g., single dose vaccination vs. campaigns every few years).

## Cholera laboratory diagnostic tools and capacity building

### Cholera diagnosis in Africa

Within the Africhol project, suspected cases of cholera should be confirmed by laboratory diagnosis whenever possible. More than half of the suspected cases had a stool sample collected, with 37% having a positive culture. Great disparities existed between countries, with positivity rates ranging from less than 30% (Kenya and Mozambique) to over 60% (Togo, Tanzania and Zimbabwe). The predominant serotypes also varied between countries, with mainly Ogawa (Guinea and Togo) in some and Inaba (DRC and Tanzania) in others. External quality control was set up at the National Institute for Communicable Diseases (NICD) in Johannesburg, South Africa. An increase in antibiotic resistance occurred during the study period, potentially due to high self-medication and the use of antibiotics for treating other diseases.

A variety of new technologies are becoming available for rapid and multiplex testing of *Vibrio cholerae.* However, they must become more accessible and affordable for use in low-resource settings.

### Laboratory capacity strengthening and technology transfer

Within the Africhol project, laboratory capacity for the confirmation of *Vibrio cholerae* was developed to obtain more precise burden estimates. We reinforced the operational capacity of the provincial laboratory in Goma, DRC (Ami Labo), thereby illustrating how high-quality bacteriological diagnostics can be adapted to challenging low-resource settings. This reinforcement resulted in a substantial increase in the number of strains analyzed in Goma, from 450 between 1998 and 2010 to 1,350 between 2011 and May 2015. Further knowledge transfer was provided in all Africhol countries. In addition, staff of AMP and the University of Gothenburg trained biologists at the consortium meeting on molecular techniques to isolate and identify *V. cholerae* in stool.

### Collection of environmental data

Environmental surveys were carried out in 2014 in enhanced surveillance sites in Lomé and Goma. Water and fish samples were taken monthly for six months. In addition, samples were taken at the home of cases during outbreak investigations. In Togo, no *V. cholerae* were identified in the environment in the absence of an epidemic, while during epidemics *V. cholerae* O1 Ogawa was identified in 4 of 6 case contacts (persons living in the same compound as the case, including case households and other households sharing the same well) and in their environment (two wells and bath water in one instance), and three *Vibrio spp* were found in (one each in a well, public restroom and drinking water). In Goma, DRC, the local laboratory found *V. cholerae* O1 Hikojima (3), non O1 non O139 (19), and O139 strains (3) in lake water and in caught Tilapia fish.

### Research on *V. cholerae* pathogen and cholera vaccines

Colleagues Lebens and Karlsson from the University of Gothenburg, Sweden, shared their experience and knowledge, co-organized the laboratory workshop and met the key actors working in cholera at the national and international level. They presented their ongoing work on differences in the observed persistence of *V. cholerae* strains in India, possibly correlated to mutations in the gene that determines the Ogawa/Inaba serotype and its implications for allowing early detection of epidemic-prone strains. They also updated the group on a current research towards simplified, cheap and low-cost cholera vaccines that could be potentially field-tested in cholera-affected countries in the future.

## Recommendations and conclusions

### Conclusions from the meeting

The implementation of this multi-country project in Africa within a consortium of institutions and countries has enabled common working standards; progress in networking, including South-South and North–South collaboration and international mobilization; improved political support and willingness to address cholera control; knowledge transfer and capacity building; and financial support and sustainability through distribution of work between partners.

Over 10,000 suspected cases have been reported and over 5,000 stool samples tested during the 5 years of surveillance. Initially, only aggregated notifications of cholera by year and country were available. Now the number of suspected and confirmed cases at neighborhood level, including morbidity and mortality, is known for specific areas, such as villages and urban neighborhoods. We believe that understanding cholera epidemiology at this level is necessary as prevention and control interventions are implemented locally and therefore national level data are of limited use in planning impactful interventions.

Another important but underappreciated dimension of cholera epidemiology – and one missing from aggregated surveillance data – is population behaviours that increase cholera risk. These behaviours may impact all interventions, including WASH and vaccine use. Finally, OCV availability represents a new opportunity for cholera control, and countries may apply for the vaccine at the ICG (for use in emergency settings) or at the GTFCC (for preemptive use).

### Recommendations for future public-health work and research activities on cholera and OCV

#### Cholera surveillance

- Improve epidemiological surveillance and laboratory capacity for cholera diagnosis (especially at regional and district levels) to better document disease burden, plan timely prevention and response measures including context-adapted WASH activities and assess the impact of future OCV immunization campaigns.
- Adapt the case definition in high-burden areas to include children age 2 years and above since many cases in Africhol countries were in the 2–5 year-old age group.
- Adapt cholera surveillance to the broader context of overlapping epidemics of different diseases, for example Ebola.
- Test new tools and technologies such as real-time case reporting.
- Continue to identify hotspots, with refinement of hotspot definitions to include the characterization of high-risk groups in addition to geographical criteria.
- Directly brief provincial and municipal authorities on the existing high-burden areas under their authority.
- Routinely assess behavioural indicators such as (seasonal) migration patterns and age and gender specific risk behaviours, at least in high-burden areas.

#### Cholera diagnostic tools

- Improve the sensitivity and specificity of existing or new RDTs, and lower their cost to make them more useful for individual diagnosis and epidemiologic studies.

#### Research on OCV use

- Conduct more studies to evaluate preventive use of OCV in endemic settings, in addition to the focus on reactive campaigns in emergency settings.
- Test simplified immunization strategies. The current guidelines recommend the administration of two doses of the Shanchol™ vaccine; a single dose strategy could be explored to determine whether it provides rapid immunity in populations that were naturally immunized or previously vaccinated [12–15].
- Evaluate OCV field effectiveness outside the cold-chain or in specific settings (schools, etc.); assess OCV safety and effectiveness in specific groups (pregnant women, travelers); and further characterize herd protection.
- Model interventions to prevent outbreaks to determine optimal strategies for different epidemiological contexts [12, 16, 17].
- Conduct cost-effectiveness analyses for low resource settings [18, 19].
- Investigate knowledge, attitudes and practices of specific high-risk groups (such as fishing communities) through anthropological studies, to adapt prevention and control strategies.

These proposed actions should be conducted through continuing and expanding North–South collaborations on specific projects with universities and non-governmental organizations working in the field of public health, research and infectious diseases, and with support from international organizations and partners, such as Gavi, BMGF, WHO (GTFCC) and bilateral aid agencies. Each country should determine its own priorities according to its political and epidemiological context. Countries should develop a multi-year plan for the prevention and control of cholera, which will also provide a road map for partners wishing to support cholera control activities.

### Recommendations for evidence-based health policy regarding cholera

- Develop guidelines on the rationale use of OCV and integrated national cholera plans for easier decision-making in preparing for immunization campaigns, in addition to context-adapted long-term WASH interventions.
- Strengthen surveillance systems including laboratory diagnostic capacity to allow early detection of epidemics and appropriate response and control.
- Train health personnel in the diagnosis, management and reporting of cases.
- Better document country cholera features and continue research on cholera and OCV.
- Have functional National Immunization Technical Advisory Groups (NITAGs) to support decisions-makers in taking evidence-based decisions on control measures including the use of vaccine.
- Strengthen cross-border collaboration between at-risk countries, to enhance joint surveillance, diagnostics and reporting of cholera and other priority cross-border health issues for mutual benefits.
- Prepare to strengthen, expand and sustain the Africhol consortium. The gains that have been made must be backed up by strong North-South cooperation via complementary projects, as well as South-South cooperation as has already been initiated.

## Additional file

Additional file 1: List of participants. (DOCX 23 kb)
