# Supplementary material for: The African cholera surveillance network (Africhol) consortium meeting, 10–11 June 2015, Lomé, Togo
Source: BMC Proc. 2017 Jan 31;11(Suppl 1):2. doi: 10.1186/s12919-016-0068-z (PMC5301166; doi:10.1186/s12919-016-0068-z)
Supplement: Additional file 1: — List of participants. (DOCX 23 kb) [file 12919_2016_68_MOESM1_ESM.docx]

**Additional File 1:**  List of participants

|  | **Participants** | **Country** | **Institution** | | **Position** |
| --- | --- | --- | --- | --- | --- |
| ***Member countries*** | | | | | |
| **1** | Lucienne Dempouo | Cameroon | Fight against epidemics and pandemics Unit, Public health Ministry | Head,  Africhol focal point | |
| **2** | Guy Vernet | Cameroon | Centre Pasteur du Cameroun | Director General | |
| **3** | Emilienne N’Guetta | Côte d’Ivoire | Institut National d’Hygiène Publique | Africhol surveillance officer | |
| **4** | Jean Claude Anné | Côte d’Ivoire | Institut Pasteur Côte d’Ivoire | Researcher at the national reference center Cholera and Shigellosis | |
| **5** | Didier Bompangue Nkoko | Democratic Republic of the Congo (DRC) | Kinshasa University | Africhol focal point | |
| **6** | Laurent Akilimali | DRC | Ministry of Health, Kinshasa | Africhol country coordinator | |
| **7** | Guy Mutombo | DRC | Ministry of Health, North Kivu province, Goma | Africhol surveillance officer | |
| **8** | Berthe Miwanda | DRC | Institut National de Recherche Biomédicale (INRB) | National laboratory coordinator | |
| **9** | Mamadou Saliou Hafia Diallo | Guinea | Laboratoire national de Santé Publique | Biologist | |
| **10** | Ian Njeru | Kenya | Disease surveillance and response Unit, Ministry of Health | Head,  Africhol focal point | |
| **11** | José Paulo Langa | Mozambique | Instituto Nacional de Saude | Microbiology laboratory manager | |
| **12** | Liliana Dengo | Mozambique | Instituto Nacional de Saude | Laboratory coordinator | |
| **13** | Akina Oyemakinde | Nigeria | Federal Ministry of Health | Chief consultant epidemiologist  Africhol focal point | |
| **14** | Olubunmi Ojo | Nigeria | Federal Ministry of Health | Director disease surveillance /International Health Regulations  Africhol country coordinator | |
| **15** | Jacob Lusekelo | Tanzania | National Health Laboratory | Country laboratory coordinator | |
| **16** | Elibariki Mwakapeje | Tanzania | Ministry of Health and Social Welfare | Coordinator, disease surveillance | |
| **17** | Neema Saul | Tanzania | Ministry of Health and Social Welfare | Laboratory technician | |
| **18** | Abiba Kere-Banla | Togo | Institut National d’Hygiène (INH) | Director,  Africhol focal point | |
| **29** | Tsidi Agbeko Tamekloe | Togo | Ministry of Health | Head, Surveillance of Communicable diseases Division | |
| **20** | Ouyi Tante | Togo | Ministry of Health | Health engineer  Africhol surveillance officer | |
| **21** | Pawou Bidjada | Togo | INH | Biotechnologist engineer | |
| **22** | Issa Zoulkarnéiri | Togo | INH | Biotechnologist engineer | |
| **23** | Aristide Komla Koba | Togo | INH | Biotechnologist engineer | |
| **24** | Koffi Semènou Awoussi | Togo | INH | Biotechnologist engineer | |
| **25** | Komlan Kossi | Togo | INH | Biotechnologist engineer | |
| **26** | Issaka Maman | Togo | INH | Biotechnologist engineer | |
| **27** | Atek Kagirita | Uganda | Ministry of Health | National laboratory coordinator | |
| **28** | Portia Manangazira | Zimbabwe | Epidemiology and disease control, Ministry of Health and Child Care | Director  Africhol focal point | |
| **29** | Isaac Phiri | Zimbabwe | Epidemiology and disease control, Ministry of Health and Child Care | Deputy Director  Africhol country coordinator | |
| **30** | Andrew Tarupiwa | Zimbabwe | National Microbiology Reference Laboratory | Principal medical laboratory scientist | |
| ***Invited non-Africhol country*** | | | | | |
| **31** | Maurice Mbangombe | Malawi | Ministry of Health | Epidemiologist | |
| ***Africhol coordination team*** | | | | | |
| **32** | Aristide Aplogan | Côte d’Ivoire | Agence de Médecine Préventive (AMP) | Representing the AMP executive director  Program leader, Field epidemiology and vaccinology | |
| **33** | Martin Mengel | France | AMP | Project director | |
| **34** | Berthe-Marie Njanpop-Lafourcade | France | AMP | Laboratory director | |
| **35** | Delphine Sauvageot | France | AMP | Project Coordinator for West Africa | |
| **36** | Bekithemba Mhlanga | Zimbabwe | AMP | Project Coordinator for East Africa | |
| **37** | Leonard Heyerdahl | Côte d’Ivoire | AMP | Project Operations Manager | |
| **38** | Richard Wood | France | AMP | Senior data analyst | |
| **39** | Johara Nadri | Côte d’Ivoire | AMP | Field epidemiologist | |
| **40** | Issaka Ouédraogo | Burkina Faso | AMP | Data manager | |
| **41** | Alexandre Blake | France | AMP | Medical epidemiologist | |
| **42** | Aline Munier | France | AMP | Pharmacist epidemiologist | |
| ***Present member institutions (Africhol)*** | | | | | |
| **43** | Sheba Nakacubo Gitta | Uganda | African Field Epidemiology Network (AFENET) | Deputy executive director and Head of Science and Public Affairs | |
| **44** | Dennis Chao | USA | Fred Hutchinson Cancer Research Center, Seattle;  Intellectual Ventures, Institute for Disease Modeling group, Bellevue, USA | Assistant researcher | |
| **45** | Eric Mintz | USA | Centers for Disease Control and Prevention (CDC), Atlanta | Team leader, Global Water, Sanitation and Hygiene Epidemiology | |
| **46** | Félicité Chokki | Burkina Faso | West-African Health Organization (WAHO), Bobo-Dioulasso | Responsible for preparation and response to epidemics | |
| **47** | Justin Lessler | USA | Johns Hopkins University, Baltimore | Assistant professor | |
| ***Other present institutions*** | | | | | |
| **48** | Michael Lebens | Sweden | University of Gothenburg  Institute of Biomedecine, Department of Microbiology and Immunology | Researcher | |
| **49** | Stefan Karlsson | Sweden | University of Gothenburg  Institute of Biomedecine, Department of Microbiology and Immunology | Researcher | |
| **50** | Marie-Laure Quilici | France | Institut Pasteur Paris,  Research and Expertise in Enteric Pathogenic Bacteria Unit | Scientist | |
| ***International Partners*** | | | | | |
| **51** | Evan Sturtevant | USA | Bill & Melinda Gates Foundation (BMGF, Seattle) | Program officer | |
| **52** | Lorenzo Pezzoli | Switzerland | Global Task Force for Cholera Control (GTFCC), Geneva | GTFCC secretariat | |
